# How Bodily Interaction Shapes Creativity: Two Experiments on Direction and Type

**DOI:** 10.3390/bs16050735

**Published:** 2026-05-09

**Authors:** Jiajia Su, Haosheng Ye

**Affiliations:** 1 School of Educational Science, Jiangsu Second Normal University, Nanjing 211200, China; sujiajia0929@163.com; 2 The Center for Embodied Cognition, Guangzhou University, Guangzhou 510006, China

**Keywords:** body interaction, creativity, embodied metaphor, enactive metaphor

## Abstract

To address the challenges in embodied creativity research, this study innovatively developed a new mirror game experimental paradigm based on the dual theoretical framework of embodied metaphor and enactive metaphor. From the perspective of embodied metaphor, the study investigates how “body interaction direction” (imitation vs. avoidance) influences delayed creativity. From the perspective of enactive metaphor, it examines how “body interaction type” (leader–follower vs. Joint Improvisation) shapes immediate creativity and reveals the overall effects of their interaction on creativity. Two experiments were conducted in this study: Experiment 1 focused on virtual human–machine interaction, and Experiment 2 explored real-life human–human interaction. Experiment 1 validated the synergistic effects of body interaction direction and type on individual creativity dimensions, with Joint Improvisation showing significant advantages. Experiment 2 further demonstrated that embodied and enactive metaphors exhibit significant synergistic effects on group creativity, with the leader–follower interaction pattern showing distinct value in the context of interaction effects. The general discussion highlights that embodied and enactive metaphors, as dual mechanisms, provide a theoretical breakthrough for understanding creativity, spanning human–machine and human–human interactions. Within the framework of enactive cognition, this study further expands the sense-making function of metaphor theory in creativity development.

## 1. Introduction

In recent years, two paradigms have gained increasing attention in cognitive science and creativity research: embodied cognition and the mirror game paradigm. Embodied cognition proposes that the body plays a fundamental role in cognition, and in creativity research, this perspective has developed into embodied creativity, emphasizing the role of bodily experience in creative thinking (Stanciu, 2015; Frith & Miller, 2024; Malinin, 2025). Meanwhile, the mirror game paradigm has emerged as a valuable framework for examining real-time bodily coordination and social interaction (Noy et al., 2011; Keisari et al., 2022; Llobera et al., 2022).

Although previous mirror game studies have demonstrated the value of manipulating interaction type (e.g., leader–follower vs. Joint Improvisation), they have mainly focused on role structure and coordination mode (Noy et al., 2011; Gueugnon et al., 2016; Keisari et al., 2022). Comparatively less attention has been given to interaction direction, namely, whether individuals coordinate through mimicry or avoidance. This omission limits the understanding of how bodily interaction patterns shape metaphor generation, sense-making, and creativity (De Jaegher & Di Paolo, 2007; Malinin, 2019; Green et al., 2024; Schiavio et al., 2024).

To address this gap, the present study extends the classic mirror game paradigm by introducing interaction direction (mirror mimicry vs. mirror avoidance) as an additional experimental factor. Combined with interaction type, this design enables a more comprehensive examination of how bodily interaction influences creativity. Accordingly, the present study tests the following hypotheses:

**Hypothesis for the Main Effects of “Bodily Interaction Direction”:**

**H1.**  *Bodily interaction direction will significantly affect multiple dimensions of creativity (fluency, flexibility, novelty, and persistence).*

**Hypothesis for the Main Effects of “Bodily Interaction Type”:**

**H2.**  *Bodily interaction* *type will significantly affect multiple dimensions of creativity.*

**Hypothesis for the Interaction Effects of Bodily Interaction Direction and Type:**

**H3.**  *Bodily interaction direction and* *type interact to shape multidimensional creativity performance.*

## 2. Materials and Methods

### 2.1. Research Aims and Overall Design

To examine the effects of bodily interaction direction and type on creativity, this study employed a parallel two-experiment design. Experiment 1, based on the mirror game paradigm and its human–machine adaptation, extended the paradigm by incorporating both mimicry and avoidance conditions to investigate how avatar interaction between humans and machine agents influences individual creativity. Experiment 2, building on the human–human mirror game paradigm, applied the same manipulation to examine how bodily interaction between individuals influences group creativity.

### 2.2. Participants

The two experiments were conducted with the approval of the leadership and the physical education department of the middle school in Guangzhou. Prior to the experiment, all participants signed informed consent forms. Upon completion of the experiment, a large number of school supplies were provided to the physical education department as gifts for the teachers to distribute to the participating students.

#### 2.2.1. Experiment 1: Participants

In the formal experimental phase, approximately 500 junior high school students from a middle school in Guangzhou participated in this study. The sample included 12 classes, each with about 40 students, and each experimental group consisted of students from two classes. Due to unforeseen circumstances during the experiment and issues with data collection post-experiment, some students’ data were incomplete and thus excluded from further analysis. Ultimately, valid data from 477 participants were included in the final analysis (83 in the Machine Imitates Human group, 90 in the Human Imitates Machine group, 74 in the Mutual Imitation group, 78 in the Machine Avoids Human group, 72 in the Human Avoids Machine group, and 80 in the Mutual Avoidance group).

#### 2.2.2. Experiment 2: Participants

During the formal experimental phase, approximately 320 middle school students from a school in Guangzhou participated in this study. The study involved eight classes, with about 40 students per class, and two classes participating in each experimental group. Due to unforeseen circumstances during the experiment and issues with data recovery after the experiment, some students’ data were incomplete and thus excluded from further analysis. Ultimately, 106 pairs of participants were included in the data analysis (26 pairs in the follower imitating leader group, 26 pairs in the Mutual Imitation group, 27 pairs in the follower avoiding leader group, and 27 pairs in the Mutual Avoidance group).

### 2.3. Experimental Design and Procedure

#### 2.3.1. Experiment 1: Design and Procedure

**Figure 1:**
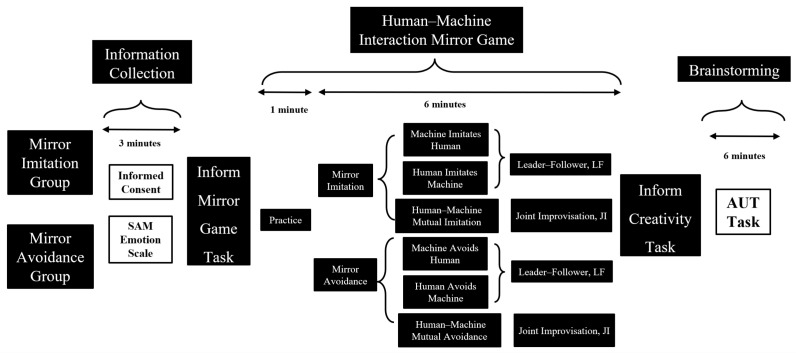
Flowchart for Experiment 1.

Experiment 1 adopted a 2 (Direction of Mirror Game Interaction) × 3 (Type of Mirror Game Interaction) between-subject design (Figure 1).

**(1) Experiment 1: Independent Variables**

**Types of Body Interaction:** The experimental conditions included the following three types of body interaction. (1) Leader Condition: Participants acted as leaders, guiding the virtual player (virtual player, VP) in performing actions, with the VP attempting to mirror the participant’s movements as accurately as possible. (2) Follower Condition: Participants acted as followers, mirroring the VP’s movements to complete the interaction. (3) Joint Improvisation Condition (Joint Improvisation, JI): No predefined roles for the leader or follower were assigned between the participant and the VP. Both dynamically adjusted their movements in a natural state, attempting to mirror each other.

**Directions of Body Interaction:** The experimental conditions further included two interaction directions. **(1) Mirror Mimicry:** Participants were required to completely match their movements with the VP’s, emphasizing coordination and consistency metaphors. **(2) Mirror Avoidance:** Participants were required to interact by performing movements opposite of the VP’s, highlighting conflict and incongruity metaphors. By combining the three types of body interaction with the two interaction directions, a total of six experimental groups were designed.

**(2) Experiment 1: Dependent Variables**

The Alternative Uses Task (AUT) was used as the creativity task. It required participants to think of as many novel and appropriate uses for everyday objects as possible, representing a classic divergent thinking task (Guilford, 1967).

In assessing creativity using the Alternative Uses Task (AUT), three evaluation dimensions are typically considered: fluency, flexibility, and novelty (Runco & Jaeger, 2012). In this study, three dimensions were assessed. **(1) Fluency:** The number of ideas generated, which was evaluated using an objective calculation method. **(2) Flexibility:** The number of different categories of ideas generated, which was assessed through subjective scoring. **(3) Novelty:** The rarity of ideas, also assessed subjectively. Specifically, two psychology graduate students with specialized training served as independent raters. They were equipped with the sufficient knowledge of creativity to ensure that their scoring consistency met the required standard. The final scores were averaged from the two raters. Based on the theoretical model of creative persistence (Nijstad et al., 2010) and its behavioral measurement framework (Wu & Koutstaal, 2022), **(4) persistence** was operationally defined as the ratio of fluency to flexibility (Persistence = Fluency/Flexibility). This metric reflects the extent to which individuals consistently generate ideas within a single conceptual category or line of thought.

**(3) Experiment 1: Additional Variables**

The Self-Assessment Manikin (SAM) scale, developed by Bradley and Lang (1994), was used to evaluate participants’ emotions. This pictorial scale calculates scores for valence, arousal, and dominance and is recognized as a classic emotional assessment tool.

#### 2.3.2. Experiment 2: Design and Procedure

**Figure 2:**
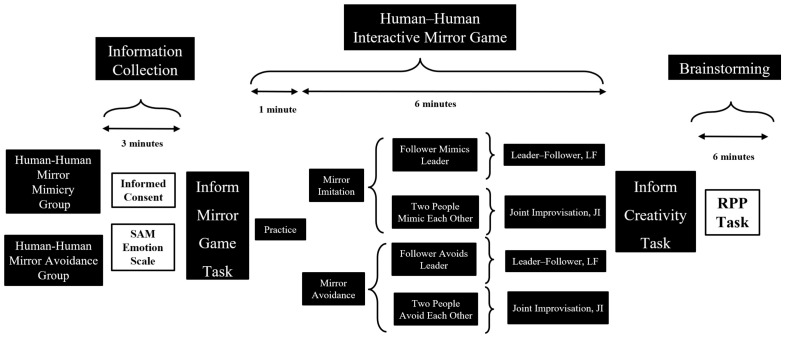
Flowchart for Experiment 2.

The second experiment adopted a 2 (Types of Interaction in the Mirror Game) × 2 (Directions of Interaction in the Mirror Game) between-subjects design (Figure 2).

**(1) Experiment 2: Independent Variables**

**Types of Interaction in the Mirror Game:** The experimental conditions included two interaction types. **(1) Leader–Follower (LF):** One participant acts as the leader to guide the other’s actions, while the other participant follows by mirroring the leader’s movements. **(2) Joint Improvisation (JI):** The dyad operates without fixed roles, freely mirroring or interacting with each other in a natural state, dynamically adjusting their movements.

**Directions of Interaction in the Mirror Game: (1) Mirror Mimicry:** Participants are required to completely mimic the actions of their partner, emphasizing coordination and consistent embodied metaphors in body interaction. **(2) Mirror Avoidance:** Participants are required to perform actions that are directly opposed to their partner’s, highlighting conflict and inconsistent embodied metaphors in body interaction.

By combining the two types of interaction (leader–follower vs. Joint Improvisation) with the two directions of interaction (mimicry vs. avoidance), the experiment formed four groups. The goal was to explore how the types and directions of body interaction influence various dimensions of group creativity through dynamic coupling mechanisms.

Participants were seated according to pre-arranged classroom seating and paired accordingly; thus, dyads were not self-selected. Given that participants completed the task in pairs and were nested within classroom contexts, potential dyadic dependence and classroom-level clustering may exist. These dependencies were not explicitly modeled in the present study and should be considered when interpreting the results.

**(2) Experiment 2: Dependent Variables**

The second experiment employed the Realistic Presented Problem (RPP) task as the creativity measure during the formal experimental phase. The RPP task, a typical divergent thinking task, assesses participants’ ability to solve open-ended, real-world problems (Runco, 1994). Participants were instructed to generate as many novel ideas as possible and were encouraged to refine and integrate ideas generated by their partners.

Given that the participants were primarily students, a real-world problem commonly encountered in their daily learning context was selected. Consistent with the AUT in Experiment 1, the RPP task was evaluated along four dimensions: **fluency, flexibility, persistence**, and **novelty**. In addition, a new dimension—**idea integration**—was introduced to assess the extent to which participants within a dyad adopted and combined each other’s ideas to generate new ones.

Fluency was assessed objectively as the total number of ideas generated by both participants within each dyad. Flexibility (i.e., the number of idea categories) and novelty (i.e., the rarity of ideas) were evaluated using subjective ratings by two trained independent raters. Each rater independently scored each participant’s responses, and the final scores were obtained by averaging the ratings from the two raters at the dyadic level. Persistence was calculated as the ratio of fluency to flexibility. Idea integration was assessed using the Consensual Assessment Technique (CAT). Two independent raters evaluated the ideas generated by each dyad before and after the interaction using a 5-point Likert scale (1 = no integration, 5 = full integration). The final integration score for each dyad was calculated by averaging the ratings from the two raters.

### 2.4. Experimental Equipment and Measures

#### 2.4.1. Experiment 1: Equipment and Measures

**Figure 3:**
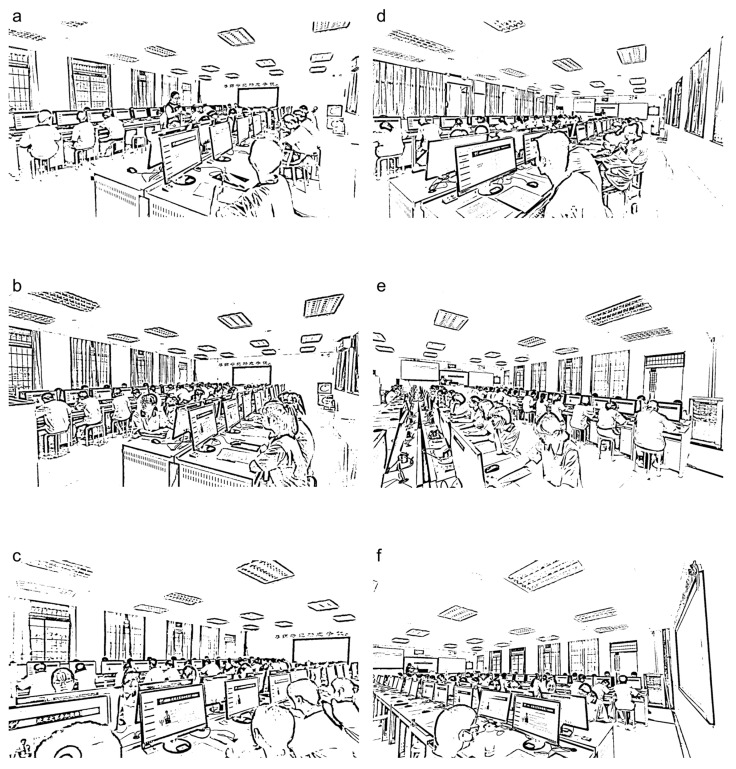
Photos of the Experiment 1 setup. Note: (**a**–**f**) Different views of the experimental setup. The Chinese text displayed on the computer screens represents the experimental program interface used for Chinese participants.

**Figure 4:**
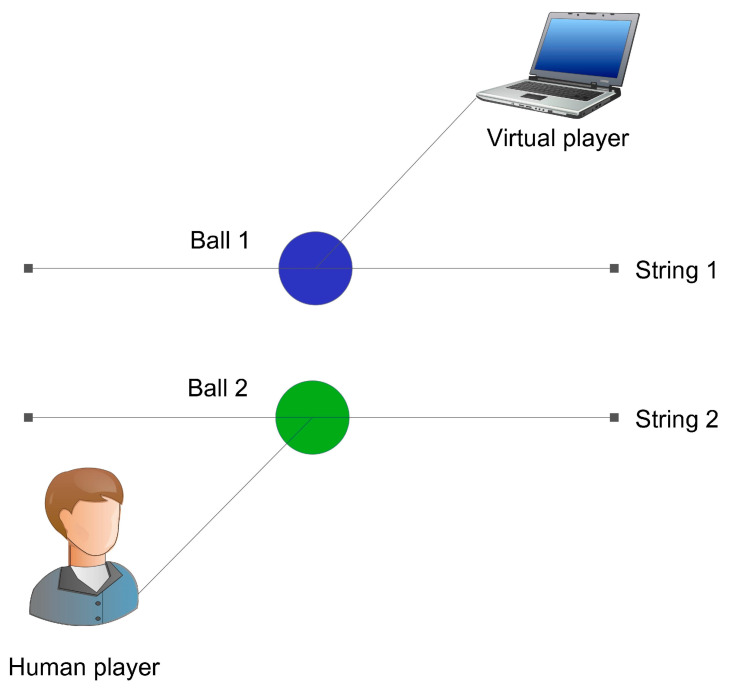
Human–Machine Interaction Mirror Game from Zhai et al. (2014).

**Figure 5:**
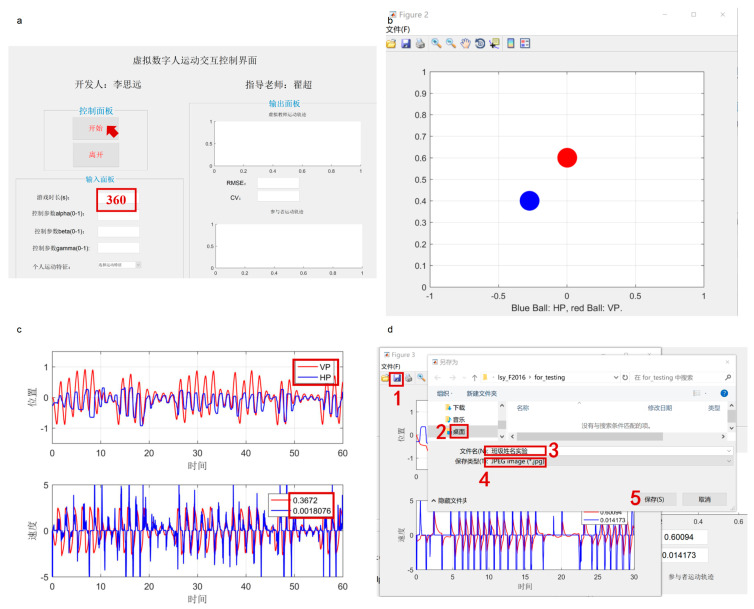
Human–Machine Interaction Mirror Game (imitation + avoidance) paradigm program (original). Note: (**a**) Main interface of the Human–Machine Interaction Mirror Game program; (**b**) the blue dot represents the human player, and the red dot represents the virtual player; and (**c**) the blue line and red line represent the movement trajectories of the human player and the virtual player; (**d**) save interface of the Human–Machine Interaction Mirror Game. The Chinese text displayed in the program interface reflects the original Chinese-language experimental software developed for Chinese middle school students and is retained for authenticity and ecological validity.

As shown in Figure 3, Experiment 1 was conducted in the computer lab of a middle school in Guangzhou. The computers were equipped with 19-inch monitors with a screen resolution of 1024 × 768 pixels. All experimental procedures in Experiment 1 were programmed and executed using Matlab (2016b), which also generated interaction trajectory graphs with RMSE and CV data for the human–machine interaction game (see Figure 4 and Figure 5).

**(1) Mirror Game Procedure**

Although Experiment 1 further adapts the Human–Machine Interaction Mirror Game (imitation + avoidance) paradigm, employing a 2 (direction of human–machine interaction) × 3 (type of human–machine interaction) between-subjects design, resulting in six groups in total, the actual Human–Machine Interaction Mirror Game (imitation + avoidance) procedure comprises only four distinct groups. Specifically, the “Mutual Imitation” group uses the same program as the “Machine Imitates Human” group, and the “Mutual Avoidance” group uses the same program as the “Machine Avoids Human” group. In other words, for the “Mutual Imitation” and “Mutual Avoidance” groups, it suffices to modify the existing machine imitation or avoidance programs by giving corresponding imitation or avoidance commands to the human players without the need to design additional programs.

**(2) VP Mathematical Model**

The Human Dynamic Clamp (HDC) framework, developed by Zhai et al. (2014, 2018), provides a real-time bidirectional interaction method, allowing for dynamic interactions between humans and computational models (such as HKB). This framework enables the real-time adjustment of model parameters to simulate specific aspects of human behavior, facilitating deeper research into the interaction dynamics between humans and virtual systems. In essence, the HKB model focuses on the theoretical framework of motor coordination, while the HDC framework applies these theories to real-time human–machine interactions.

Given the large sample size in this study (nearly 500 participants), this study adopts the HDC framework as the VP model.

**(3) Quantitative Interaction Metrics**

Consistent with the metrics used in the study by Li and Zhai (2022), this research introduces two performance metrics to quantify the impact of the human player (HP) on the virtual player (VP) under teaching conditions: Root Mean Square Error (RMSE) of the position time series and Circular Variance (CV).

*RMSE* is used to describe the spatial synchronization of the movements of the two interaction participants, where (*n*) is the total number of sampling steps in the experiment, and (*X*_1,*j*_) and (*X*_2,*j*_) represent the positions of the two players at the (*j*)-th sampling step.

$$RMSE=\sqrt{\frac{1}{n}\sum_{j=1}^{n}(X_{1,j}-X_{2,j}{)}^{2}}$$

*CV* is used to describe the level of phase constancy between the two players, where (∆Φ_*j*_) represents the relative phase of the two players at the (*j*)-th sampling step, and (*n*) is the total number of sampling steps. The term (*e*^*i*∆Φ_*j*_^) is the exponential function with base (*e*) used to calculate the phase part of the complex number, thereby assessing the phase synchronization between the two movements.

$$CV=\left\|\frac{1}{n}\sum_{j=1}^{n}e^{i\Delta \Phi_{j}}\right\|$$

#### 2.4.2. Experiment 2: Equipment and Measures

**Figure 6:**
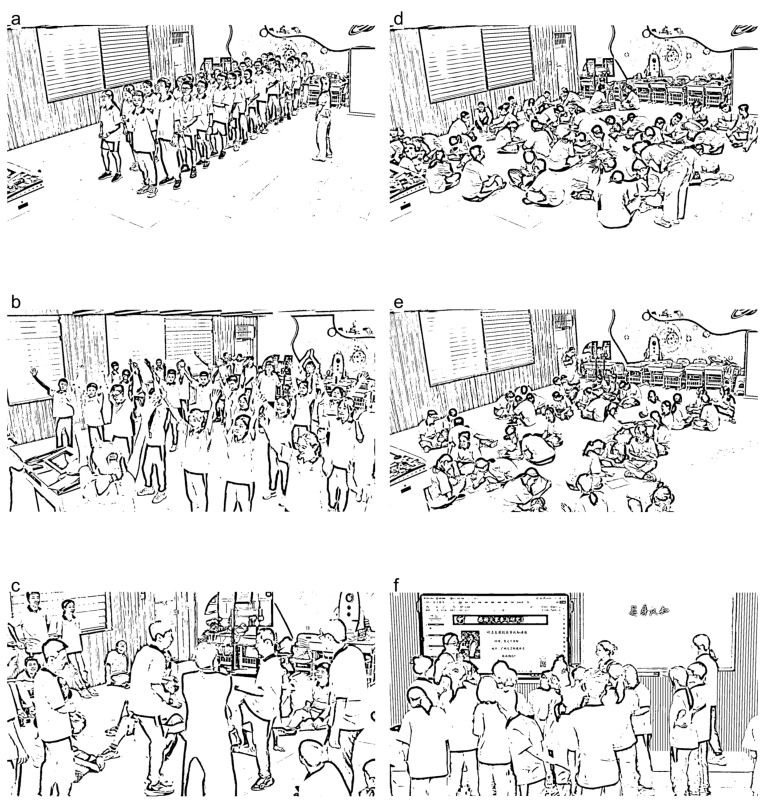
Photos of the Experiment 2 setup. Note: (**a**–**f**) Different views of the experimental setup. The Chinese text displayed on the computer screens represents the experimental program interface used for Chinese participants.

As shown in Figure 6, Experiment 2 was conducted with four groups (eight classes) in the smart classroom K205 of a middle school in Guangzhou. Built in June 2020, the classroom is equipped with iFlytek tablets to support dynamic teaching and evaluation. It also features an Aoweiya recording system, enabling one-click recording and real-time streaming. The experiment was conducted as an open class using the classroom’s teaching and recording facilities.

## 3. Results

Data were analyzed using IBM SPSS Statistics 26 and R 4.5.1, with individuals as the unit of analysis in Experiment 1 and dyads in Experiment 2.

### 3.1. Manipulation Check

#### Experiment 1: Interaction Degree

**(1) RMSE and CV: Two-way ANOVA with 3 (Type of Interaction) × 2 (Direction of Interaction) Design**

**Figure 7:**
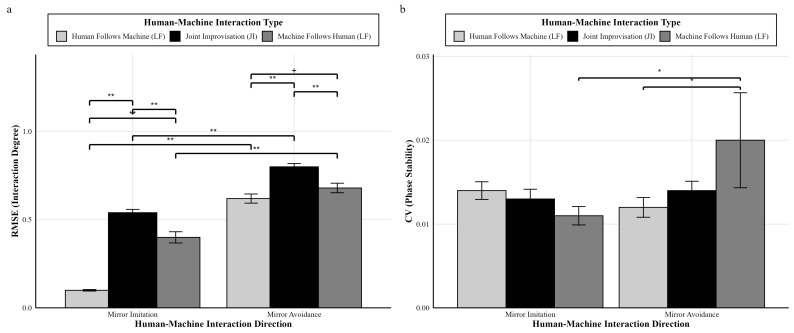
Statistical results of human–machine mirror game interaction degree (RMSE and CV). Note: (**a**) 3 × 2 ANOVA results for RMSE; (**b**) 3 × 2 ANOVA results for CV. * *p* < 0.05; ** *p* < 0.01; + *p* < 0.10.

Based on the descriptive statistics (Table 1), a two-way ANOVA was conducted to examine the effects of interaction type and interaction direction on the performance metrics (RMSE and CV). The two-way ANOVA analysis results are shown in Figure 7.

For the RMSE (Root Mean Square Error), the results indicated significant main effects for both the direction of human–machine interaction, *F*(1,414) = 328.70, *p* < 0.001, and η_p_^2^ = 0.44, and the type of human–machine interaction, *F*(2,414) = 90.22, *p* < 0.001, and η_p_^2^ = 0.30. There was also a significant interaction between the interaction direction and interaction type, *F*(2,414) = 18.89, *p* < 0.001, and η_p_^2^ = 0.08. Further simple effects analysis showed that, at the level of interaction type (Machine Follows Human, Human Follows Machine, and Joint Improvisation), the mirror imitation groups (*M* = 0.40, *SD* = 0.29; *M* = 0.10, *SD* = 0.04; and *M* = 0.54, *SD* = 0.16) had significantly lower RMSE values compared to the mirror avoidance groups (*M* = 0.68, *SD* = 0.24, and Cohen’s *d* = −1.05; *M* = 0.62, *SD* = 0.22, and Cohen’s *d* = 3.29; and *M* = 0.80, *SD* = 0.16, and Cohen’s *d* = 1.63), *p* < 0.001. At the level of the interaction direction (mirror imitation), the Machine Follows Human group (*M* = 0.40, *SD* = 0.29) had a significantly higher RMSE than the Human Follows Machine group (*M* = 0.10, *SD* = 0.04, and Cohen’s *d* = 1.45), and the Joint Improvisation group (*M* = 0.54, *SD* = 0.16) had a significantly higher RMSE than both the Machine Follows Human group (*M* = 0.40, *SD* = 0.29, and Cohen’s *d* = 0.60) and the Human Follows Machine group (*M* = 0.10, *SD* = 0.04, and Cohen’s *d* = 3.77), *p* < 0.001. For the mirror avoidance groups, the Machine Follows Human group (*M* = 0.68, *SD* = 0.24) had a marginally significantly higher RMSE than the Human Follows Machine group (*M* = 0.62, *SD* = 0.22, and Cohen’s *d* = 0.26), *p* = 0.057. The Joint Improvisation group (*M* = 0.80, *SD* = 0.16) had a significantly higher RMSE than both the Machine Follows Human group (*M* = 0.68, *SD* = 0.24, and Cohen’s *d* = 0.59) and the Human Follows Machine group (*M* = 0.62, *SD* = 0.22, and Cohen’s *d* = 0.94), *p* < 0.001.

For the CV (Circular Variance), the results indicated no significant main effect for the direction of human–machine interaction, *F*(1,414) = 1.03, *p* = 0.311, and η_p_^2^ = 0.00, and no significant main effect for the type of human–machine interaction, *F*(2,414) = 0.36, *p* = 0.695, and η_p_^2^ = 0.00. The interaction between the interaction direction and interaction type was non-significant, *F*(2,414) = 2.25, *p* = 0.107, and η_p_^2^ = 0.01. Further simple effects analysis showed that, at the level of interaction type (Machine Follows Human), the mirror imitation group (*M* = 0.01, *SD* = 0.01) had a significantly lower CV compared to the mirror avoidance group (*M* = 0.02, *SD* = 0.05, and Cohen’s *d* = 0.28), *p* = 0.027. At the level of interaction direction (mirror imitation), pairwise comparisons among all three groups—Machine Follows Human (*M* = 0.01, *SD* = 0.01), Human Follows Machine (*M* = 0.01, *SD* = 0.01), and Joint Improvisation (*M* = 0.01, *SD* = 0.01)—showed no significant differences. For the mirror avoidance groups, the Machine Follows Human group (*M* = 0.02, *SD* = 0.05) had a significantly higher CV than the Human Follows Machine group (*M* = 0.01, *SD* = 0.01, and Cohen’s *d* = 0.28), *p* = 0.041.

### 3.2. Consistency Check for Scoring

#### 3.2.1. Experiment 1: Scoring Consistency

In this study, the consistency of scoring was assessed using Cronbach’s alpha. The analysis evaluated the inter-rater reliability between two raters on the dimensions of flexibility, novelty, and persistence in the AUT (Table 2).

In general, a reliability coefficient of 0.70 or higher is considered acceptable for inter-rater reliability. The results indicate that most Cronbach’s alpha coefficients reached acceptable levels, although the novelty score for the key task (α = 0.683) was slightly below the threshold and should be interpreted with caution. It should also be noted that Cronbach’s alpha reflects internal consistency rather than absolute agreement between raters. Overall, the inter-rater reliability can be considered acceptable.

#### 3.2.2. Experiment 2: Scoring Consistency

Cronbach’s alpha was used to test the consistency of the ratings between the two raters (Table 3).

The Cronbach’s alpha coefficients for flexibility, novelty, and persistence all reached acceptable levels (α > 0.70), indicating good inter-rater reliability.

### 3.3. Dependent Variable: Performance in the Creativity Task

#### 3.3.1. Experiment 1: AUT Performance

**(1) Dimensions of Fluency, Flexibility, Novelty, and Persistence: Two-way ANOVA with 3 (Type of Interaction) × 2 (Direction of Interaction) Design**

**Figure 8:**
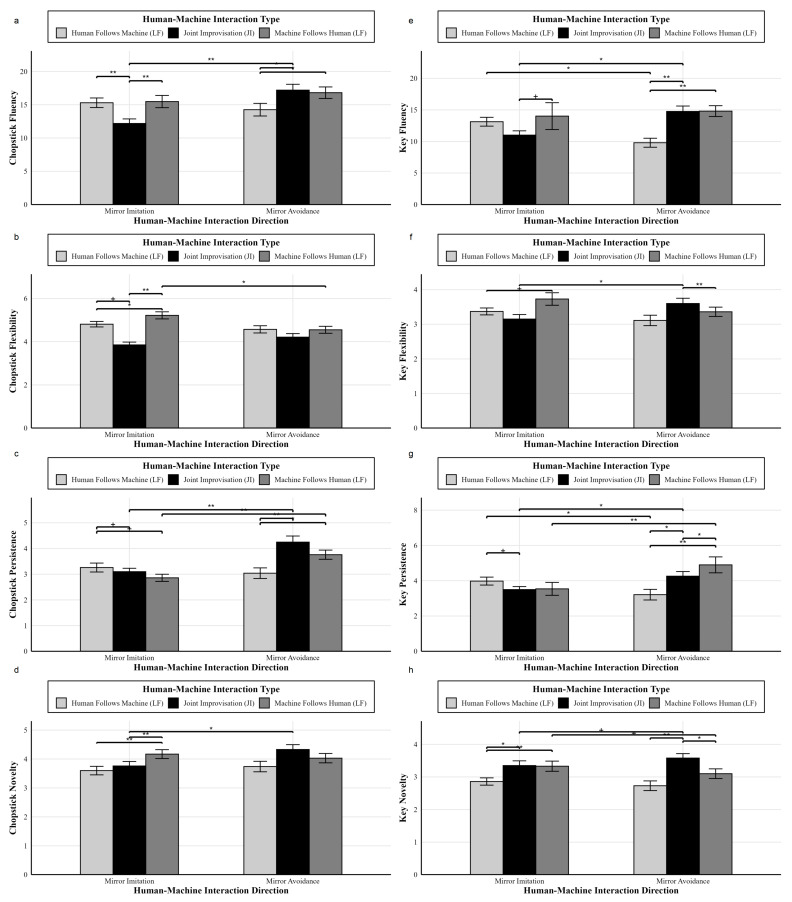
Statistical results of the chopstick and key AUTs (fluency, flexibility, persistence, and novelty). Note: (**a**–**d**) represent the 3 × 2 ANOVA results for chopstick fluency, flexibility, persistence, and novelty, respectively; (**e**–**h**) represent the 3 × 2 ANOVA results for key fluency, flexibility, persistence, and novelty, respectively. * *p* < 0.05; ** *p* < 0.01; + *p* < 0.10.

Based on the descriptive statistics (Table 4 and Table 5), a two-way ANOVA was conducted on the AUT performance (fluency, flexibility, persistence, and novelty for chopsticks and keys) with a 3 (Type of Interaction) × 2 (Direction of Interaction) design. The two-way ANOVA results are shown in Figure 8.

For the fluency with chopsticks, there was a significant main effect of the direction of human–machine interaction, *F*(1,465) = 6.10, *p* = 0.014, and η_p_^2^ = 0.01; a non-significant main effect of the type of human–machine interaction, *F*(2,465) = 1.86, *p* = 0.157, and η_p_^2^ = 0.01; and a significant interaction between the direction and type of human–machine interaction, *F*(2,465) = 6.65, *p* = 0.001, and η_p_^2^ = 0.03. Further simple effects analysis revealed that, under the condition of human–machine interaction direction (mirror imitation), the Joint Improvisation group (*M* = 12.19, *SD* = 5.86) had a significantly lower fluency than the Machine Follows Human group (*M* = 15.48, *SD* = 8.39, and Cohen’s *d* = 0.45) and the Human Follows Machine group (*M* = 15.30, *SD* = 6.82, and Cohen’s *d* = 0.49), *p* = 0.006. Under the condition of human–machine interaction direction (mirror avoidance), the Human Follows Machine group (*M* = 14.26, *SD* = 8.05) had a significantly lower fluency than the Machine Follows Human group (*M* = 16.81, *SD* = 7.65, and Cohen’s *d* = 0.32), *p* = 0.037, and a significantly lower fluency than the Joint Improvisation group (*M* = 17.20, *SD* = 7.87, and Cohen’s *d* = 0.37), *p* = 0.018. Under the condition of human–machine interaction type (Joint Improvisation), the mirror imitation condition (*M* = 12.19, *SD* = 5.86) had significantly lower fluency than the mirror avoidance condition (*M* = 17.20, *SD* = 7.87, and Cohen’s *d* = 0.72), *p* < 0.001.

For the fluency with keys, there was no significant main effect of the direction of human–machine interaction, *F*(1,465) = 0.13, *p* = 0.722, and η_p_^2^ = 0.00; there was a significant main effect of the type of human–machine interaction, *F*(2,465) = 3.25, *p* = 0.040, and η_p_^2^ = 0.01; and a significant interaction between the direction and type of human–machine interaction, *F*(2,465) = 5.08, *p* = 0.007, and η_p_^2^ = 0.02. Further simple effects analysis revealed that, under the condition of human–machine interaction direction (mirror imitation), the Machine Follows Human group (*M* = 14.02, *SD* = 19.36) was marginally significantly higher than the Joint Improvisation group (*M* = 11.01, *SD* = 5.79, and Cohen’s *d* = 0.21), *p* = 0.064. Under the condition of human–machine interaction direction (mirror avoidance), the Human Follows Machine group (*M* = 9.80, *SD* = 6.05) had a significantly lower fluency than both the Machine Follows Human group (*M* = 14.81, *SD* = 7.58, and Cohen’s *d* = 0.73) and the Joint Improvisation group (*M* = 14.76, *SD* = 7.66, and Cohen’s *d* = 0.72), *p* = 0.003. Under the condition of human–machine interaction type (Human Follows Machine), the mirror imitation condition (*M* = 13.13, *SD* = 6.64) had a significantly higher fluency than the mirror avoidance condition (*M* = 9.80, *SD* = 6.05, and Cohen’s *d* = 0.52), *p* = 0.028. Under the condition of human–machine interaction type (Joint Improvisation), the mirror imitation condition (*M* = 11.01, *SD* = 5.79) had a significantly lower fluency than the mirror avoidance condition (*M* = 14.76, *SD* = 7.66, and Cohen’s *d* = 0.55), *p* = 0.024.

For flexibility with chopsticks, there was no significant main effect of the direction of human–machine interaction, *F*(1,465) = 0.08, *p* = 0.776, and η_p_^2^ = 0.00; there was a significant main effect of the type of human–machine interaction, *F*(2,465) = 4.63, *p* = 0.010, and η_p_^2^ = 0.02; and a significant interaction between the direction and type of human–machine interaction, *F*(2,465) = 3.75, *p* = 0.024, and η_p_^2^ = 0.02. Further simple effects analysis revealed that, under the condition of human–machine interaction direction (mirror imitation), the Machine Follows Human group (*M* = 5.22, *SD* = 1.50) had a significantly higher flexibility than the Human Follows Machine group (*M* = 4.81, *SD* = 1.21, and Cohen’s *d* = 0.30), *p* = 0.023; the Human Follows Machine group (*M* = 4.81, *SD* = 1.21) was marginally significantly higher than the Joint Improvisation group (*M* = 3.85, *SD* = 1.11, and Cohen’s *d* = 0.83), *p* = 0.082; and the Machine Follows Human group (*M* = 5.22, *SD* = 1.50) had a significantly higher flexibility than the Joint Improvisation group (*M* = 3.85, *SD* = 1.11, and Cohen’s *d* = 1.04), *p* < 0.001. Under the condition of human–machine interaction type (Machine Follows Human), the mirror imitation group (*M* = 5.22, *SD* = 1.50) had a significantly higher flexibility than the mirror avoidance group (*M* = 4.55, *SD* = 1.43, and Cohen’s *d* = 0.46), *p* = 0.040.

For flexibility with keys, there was no significant main effect of the direction of human–machine interaction, *F*(1,465) = 0.13, *p* = 0.716, and η_p_^2^ = 0.00; the main effect of the type of human–machine interaction was marginally significant, *F*(2,465) = 2.67, *p* = 0.070, and η_p_^2^ = 0.01; and there was a significant interaction between the direction and type of human–machine interaction, *F*(2,465) = 3.29, *p* = 0.038, and η_p_^2^ = 0.01. Further simple effects analysis revealed that, under the condition of human–machine interaction direction (mirror imitation), the Machine Follows Human group (*M* = 3.73, *SD* = 1.65) was marginally significantly higher than the Human Follows Machine group (*M* = 3.37, *SD* = 0.95, and Cohen’s *d* = 0.27), *p* = 0.080. Under the condition of human–machine interaction direction (mirror avoidance), the Machine Follows Human group (*M* = 3.36, *SD* = 1.19) had significantly lower flexibility than the Joint Improvisation group (*M* = 3.60, *SD* = 1.36, and Cohen’s *d* = 0.19), *p* = 0.006. Under the condition of human–machine interaction type (Joint Improvisation), the mirror imitation group (*M* = 3.15, *SD* = 1.12) had significantly lower flexibility than the mirror avoidance group (*M* = 3.60, *SD* = 1.36, and Cohen’s *d* = 0.36), *p* = 0.028.

For the persistence with chopsticks, there was a significant main effect of the direction of human–machine interaction, *F*(1,465) = 15.32, *p* < 0.001, and η_p_^2^ = 0.03; no significant main effect of the type of human–machine interaction, *F*(2,465) = 0.82, *p* = 0.441, and η_p_^2^ = 0.00; and a significant interaction between the direction and type of human–machine interaction, *F*(2,465) = 8.24, *p* < 0.001, and η_p_^2^ = 0.03. Further simple effects analysis revealed that, under the condition of human–machine interaction direction (mirror imitation), the Machine Follows Human group (*M* = 2.86, *SD* = 1.27) was marginally significantly lower than the Human Follows Machine group (*M* = 3.26, *SD* = 1.65, and Cohen’s *d* = 0.27), *p* = 0.086; the Human Follows Machine group (*M* = 3.26, *SD* = 1.65) was marginally significantly higher than the Joint Improvisation group (*M* = 3.10, *SD* = 1.13, and Cohen’s *d* = 0.11), *p* = 0.066. Under the condition of human–machine interaction direction (mirror avoidance), the Machine Follows Human group (*M* = 3.76, *SD* = 1.58) had a significantly higher persistence than the Human Follows Machine group (*M* = 3.04, *SD* = 1.75, and Cohen’s *d* = 0.43), *p* = 0.010; the Human Follows Machine group (*M* = 3.04, *SD* = 1.75) had a significantly lower persistence than the Joint Improvisation group (*M* = 4.25, *SD* = 2.12, and Cohen’s *d* = 0.62), *p* < 0.001. Under the condition of human–machine interaction type (Machine Follows Human), the mirror imitation group (*M* = 2.86, *SD* = 1.27) had a significantly lower persistence than the mirror avoidance group (*M* = 3.76, *SD* = 1.58, and Cohen’s *d* = 0.63), *p* = 0.001. Under the condition of human–machine interaction type (Joint Improvisation), the mirror imitation group (*M* = 3.10, *SD* = 1.13) had a significantly lower persistence than the mirror avoidance group (*M* = 4.25, *SD* = 2.12, and Cohen’s *d* = 0.67), *p* < 0.001.

For the persistence with keys, there was a marginally significant main effect of the direction of human–machine interaction, *F*(1,465) = 3.63, *p* = 0.057, and η_p_^2^ = 0.01; a significant main effect of the type of human–machine interaction, *F*(2,465) = 3.35, *p* = 0.036, and η_p_^2^ = 0.01; and a significant interaction between the direction and type of human–machine interaction, *F*(2,465) = 7.79, *p* < 0.001, and η_p_^2^ = 0.03. Further simple effects analysis revealed that, under the condition of human–machine interaction direction (mirror imitation), the Human Follows Machine group (*M* = 3.98, *SD* = 2.14) was marginally significantly higher than the Joint Improvisation group (*M* = 3.50, *SD* = 1.44, and Cohen’s *d* = 0.26), *p* = 0.075. Under the condition of human–machine interaction direction (mirror avoidance), the Machine Follows Human group (*M* = 4.90, *SD* = 3.99) had a significantly higher persistence than the Human Follows Machine group (*M* = 3.21, *SD* = 2.56, and Cohen’s *d* = 0.51), *p* < 0.001; the Machine Follows Human group (*M* = 4.90, *SD* = 3.99) also had a significantly higher persistence than the Joint Improvisation group (*M* = 4.26, *SD* = 2.33, and Cohen’s *d* = 0.20), *p* = 0.044. The Human Follows Machine group (*M* = 3.21, *SD* = 2.56) had a significantly lower persistence than the Joint Improvisation group (*M* = 4.26, *SD* = 2.33, and Cohen’s *d* = 0.43), *p* = 0.023. Under the condition of human–machine interaction type (Machine Follows Human), the mirror imitation group (*M* = 3.54, *SD* = 3.34) had a significantly lower persistence than the mirror avoidance group (*M* = 4.90, *SD* = 3.99, and Cohen’s *d* = 0.37), *p* = 0.001. Under the condition of human–machine interaction type (Human Follows Machine), the mirror imitation group (*M* = 3.98, *SD* = 2.14) had a significantly higher persistence than the mirror avoidance group (*M* = 3.21, *SD* = 2.56, and Cohen’s *d* = 0.33), *p* = 0.042. Under the condition of human–machine interaction type (Joint Improvisation), the mirror imitation group (*M* = 3.50, *SD* = 1.44) had a significantly lower persistence than the mirror avoidance group (*M* = 4.26, *SD* = 2.33, and Cohen’s *d* = 0.40), *p* = 0.043.

For novelty with chopsticks, there was no significant main effect of the direction of human–machine interaction, *F*(1,465) = 0.97, *p* = 0.325, and η_p_^2^ = 0.00; the main effect of the type of human–machine interaction was marginally significant, *F*(2,465) = 2.91, *p* = 0.055, and η_p_^2^ = 0.01; and there was a significant interaction between the direction and type of human–machine interaction, *F*(2,465) = 4.93, *p* = 0.008, and η_p_^2^ = 0.02. Further simple effects analysis revealed that, under the condition of human–machine interaction direction (mirror imitation), the Machine Follows Human group (*M* = 4.17, *SD* = 1.40) had a significantly higher novelty than the Human Follows Machine group (*M* = 3.60, *SD* = 1.40, and Cohen’s *d* = 0.41), *p* = 0.001; the Machine Follows Human group (*M* = 4.17, *SD* = 1.40) also had a significantly higher novelty than the Joint Improvisation group (*M* = 3.76, *SD* = 1.32, and Cohen’s *d* = 0.30), *p* = 0.004. Under the condition of human–machine interaction direction (mirror avoidance), the Human Follows Machine group (*M* = 3.74, *SD* = 1.54) was non-significantly lower than the Joint Improvisation group (*M* = 4.33, *SD* = 1.49, and Cohen’s *d* = 0.39), *p* = 0.104. Under the condition of human–machine interaction type (Machine Follows Human), the mirror imitation group (*M* = 4.17, *SD* = 1.40) was marginally significantly higher than the mirror avoidance group (*M* = 4.03, *SD* = 1.43, and Cohen’s *d* = 0.10), *p* = 0.062. Under the condition of human–machine interaction type (Joint Improvisation), the mirror imitation group (*M* = 3.76, *SD* = 1.32) had a significantly lower novelty than the mirror avoidance group (*M* = 4.33, *SD* = 1.49, and Cohen’s *d* = 0.40), *p* = 0.014.

For novelty with keys, there was no significant main effect of the direction of human–machine interaction, *F*(1,465) = 0.01, *p* = 0.922, and η_p_^2^ = 0.00; there was a significant main effect of the type of human–machine interaction, *F*(2,465) = 10.32, *p* < 0.001, and η_p_^2^ = 0.04; and the interaction between the direction and type of human–machine interaction was marginally significant, *F*(2,465) = 3.00, *p* = 0.051, and η_p_^2^ = 0.01. Further simple effects analysis revealed that, under the condition of human–machine interaction direction (mirror imitation), the Machine Follows Human group (*M* = 3.33, *SD* = 1.42) had a significantly higher novelty than the Human Follows Machine group (*M* = 2.86, *SD* = 1.06, and Cohen’s *d* = 0.38), *p* = 0.001; the Human Follows Machine group (*M* = 2.86, *SD* = 1.06) had a significantly lower novelty than the Joint Improvisation group (*M* = 3.35, *SD* = 1.24, and Cohen’s *d* = 0.42), *p* = 0.018. Under the condition of human–machine interaction direction (mirror avoidance), the Machine Follows Human group (*M* = 3.10, *SD* = 1.31) had a significantly lower novelty than the Joint Improvisation group (*M* = 3.58, *SD* = 1.21, and Cohen’s *d* = 0.38), *p* = 0.011. The Human Follows Machine group (*M* = 2.73, *SD* = 1.26) had a significantly lower novelty than the Joint Improvisation group (*M* = 3.58, *SD* = 1.21, and Cohen’s *d* = 0.69), *p* < 0.001. Under the condition of human–machine interaction type (Machine Follows Human), the mirror imitation group (*M* = 3.33, *SD* = 1.42) was marginally significantly higher than the mirror avoidance group (*M* = 3.10, *SD* = 1.31, and Cohen’s *d* = 0.17), *p* = 0.080. Under the condition of human–machine interaction type (Joint Improvisation), the mirror imitation group (*M* = 3.35, *SD* = 1.24) was marginally significantly lower than the mirror avoidance group (*M* = 3.58, *SD* = 1.21, and Cohen’s *d* = 0.19), *p* = 0.089.

#### 3.3.2. Experiment 2: RPP Task Performance

**(1) Dyadic fluency, flexibility, novelty, persistence, and integrativity of ideas: a 2 × 2 two-way ANOVA analysis**

**Figure 9:**
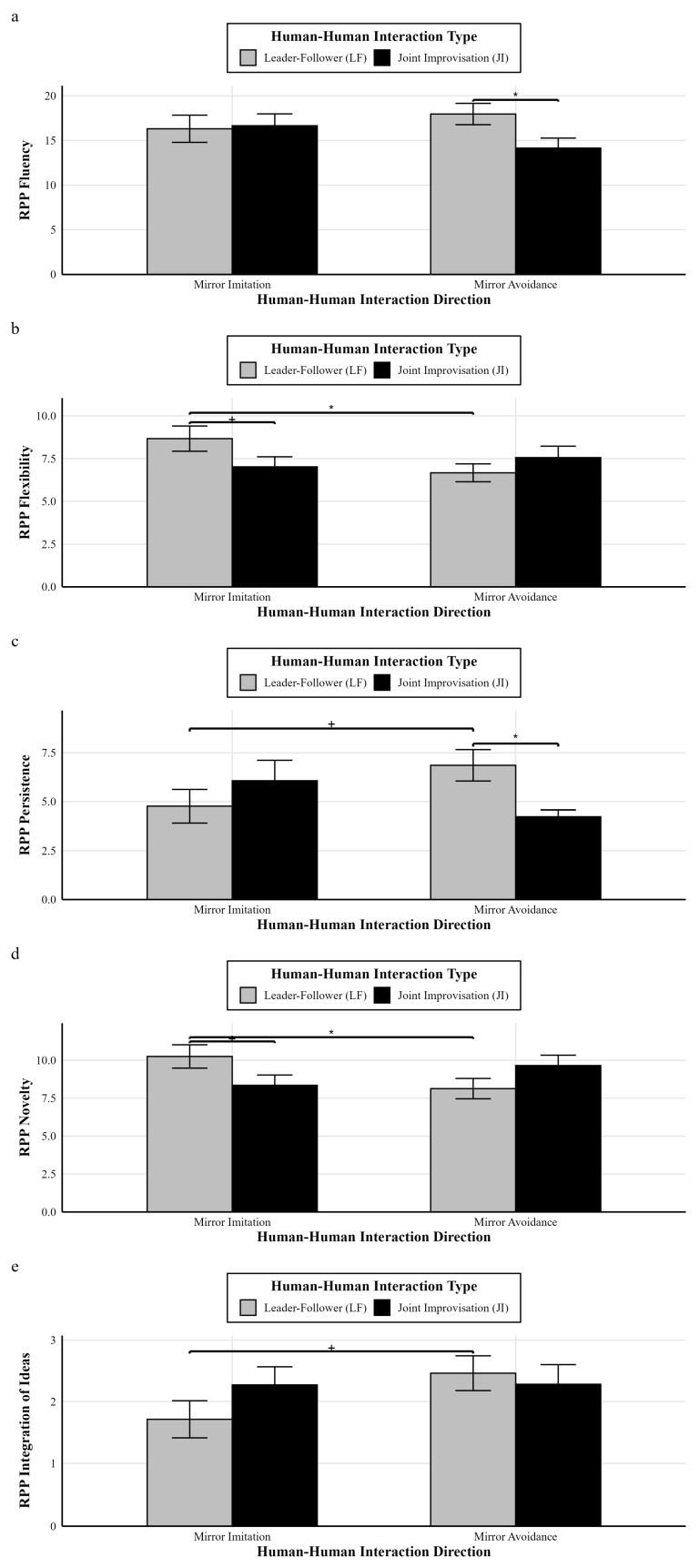
Statistical results of the RPP task (dyadic fluency, flexibility, persistence, novelty, and viewpoint integration). Note: (**a**–**d**) represent the 2 × 2 ANOVA results for dyadic fluency, flexibility, persistence, and novelty in the RPP task; (**e**) represents the 2 × 2 ANOVA results for dyadic viewpoint integration in the RPP task. * *p* < 0.05; + *p* < 0.10.

Based on the descriptive statistical results (Table 6), a 2 × 2 two-way ANOVA was conducted with the group (“Mutual Avoidance group,” “follower avoiding leader group,” “Mutual Imitation group,” and “follower imitating leader group”) as the between-subjects variable to analyze the RPP task performance (RPP fluency). The two-way ANOVA results are shown in Figure 9.

For the RPP fluency, the results show that the main effect of the human–human interaction direction was not significant, *F*(1,102) = 0.11, *p* = 0.743, and η_p_^2^ = 0.00; the main effect of the type of human–human interaction was not significant, *F*(1,102) = 1.79, *p* = 0.183, and η_p_^2^ = 0.02; and the interaction effect between the human–human interaction direction and the type of human–human interaction was not significant, *F*(1,102) = 2.58, *p* = 0.111, and η_p_^2^ = 0.03, although a trend toward an interaction was observed. Further simple effects analysis showed that, under the condition of human–human interaction direction (mirror avoidance), the follower imitating leader group (*M* = 17.96, *SD* = 6.21) was significantly higher than the joint interaction group (*M* = 14.15, *SD* = 5.80, and Cohen’s *d* = 0.64), *p* = 0.038.

For the RPP flexibility, the results show that the main effect of the human–human interaction direction was not significant, *F*(1,102) = 1.35, *p* = 0.248, and η_p_^2^ = 0.01; the main effect of the type of human–human interaction was not significant, *F*(1,102) = 0.37, *p* = 0.547, and η_p_^2^ = 0.00; and the interaction effect between the human–human interaction direction and the type of human–human interaction was significant, *F*(1,102) = 4.04, *p* = 0.047, and η_p_^2^ = 0.04. Simple effects analysis showed that, under the condition of human–human interaction direction (mirror imitation), the follower imitating leader group (*M* = 8.67, *SD* = 3.76) was marginally significantly higher than the joint interaction group (*M* = 7.02, *SD* = 3.00, and Cohen’s *d* = 0.49), *p* = 0.070; under the condition of human–human interaction type (leader–follower), the mirror imitation group (*M* = 8.67, *SD* = 3.76) was significantly higher than the mirror avoidance group (*M* = 6.67, *SD* = 2.72, and Cohen’s *d* = 0.61), *p* = 0.027.

For the RPP persistence, the results show that the main effect of the human–human interaction direction was not significant, *F*(1,102) = 0.03, *p* = 0.871, and η_p_^2^ = 0.00; the main effect of the type of human–human interaction was not significant, *F*(1,102) = 0.66, *p* = 0.411, and η_p_^2^ = 0.01; and the interaction effect between the human–human interaction direction and the type of human–human interaction was significant, *F*(1,102) = 6.01, *p* = 0.016, and η_p_^2^ = 0.06. Simple effects analysis showed that, under the condition of human–human interaction direction (mirror avoidance), the follower imitating leader group (*M* = 6.86, *SD* = 4.18) was significantly higher than the joint interaction group (*M* = 4.24, *SD* = 1.78, and Cohen’s *d* = 0.79), *p* = 0.021; under the condition of human–human interaction type (leader–follower), the mirror imitation group (*M* = 4.77, *SD* = 4.36) was marginally significantly lower than the mirror avoidance group (*M* = 6.86, *SD* = 4.18, and Cohen’s *d* = 0.49), *p* = 0.067; under the condition of human–human interaction type (joint interaction), the mirror imitation group (*M* = 6.07, *SD* = 5.34) showed a higher mean value than the mirror avoidance group (*M* = 4.24, *SD* = 1.78, Cohen’s *d* = 0.46), but the difference was not statistically significant, *p* = 0.109.

For the RPP novelty, the results show that the main effect of the human–human interaction direction was not significant, *F*(1,102) = 0.34, *p* = 0.560, and η_p_^2^ = 0.00; the main effect of the type of human–human interaction was not significant, *F*(1,102) = 0.08, *p* = 0.784, and η_p_^2^ = 0.00; the interaction effect between the human–human interaction direction and the type of human–human interaction was significant, *F*(1,102) = 5.98, *p* = 0.016, and η_p_^2^ = 0.06. Simple effects analysis showed that, under the condition of human–human interaction direction (mirror imitation), the follower imitating leader group (*M* = 10.25, *SD* = 3.92) was marginally significantly higher than the joint interaction group (*M* = 8.35, *SD* = 3.45, and Cohen’s *d* = 0.51), *p* = 0.059; under the condition of human–human interaction type (leader–follower), the mirror imitation group (*M* = 10.25, *SD* = 3.92) was significantly higher than the mirror avoidance group (*M* = 8.13, *SD* = 3.48, and Cohen’s *d* = 0.57), *p* = 0.035.

For the RPP integrativity of ideas, the results show that the main effect of the human–human interaction direction was not significant, *F*(1,102) = 1.60, *p* = 0.206, and η_p_^2^ = 0.02; the main effect of the type of human–human interaction was not significant, *F*(1,102) = 0.39, *p* = 0.536, and η_p_^2^ = 0.00; and the interaction effect between the human–human interaction direction and the type of human–human interaction was not significant, *F*(1,102) = 1.53, *p* = 0.219, and η_p_^2^ = 0.02. Simple effects analysis showed that, under the condition of human–human interaction type (leader–follower), the mirror imitation group (*M* = 1.71, *SD* = 1.54) was marginally significantly lower than the mirror avoidance group (*M* = 2.46, *SD* = 1.47, and Cohen’s *d* = 0.50), *p* = 0.080.

### 3.4. Additional Variable: Emotion

#### 3.4.1. Experiment 1: Emotion

**(1) Three dimensions of emotion (pleasure, arousal, and dominance): two-way ANOVA with 3 (Type of Interaction) × 2 (Direction of Interaction) design**

To investigate whether emotion had an impact on the experimental results, a two-way ANOVA was conducted on the three dimensions of emotion (pleasure, arousal, and dominance). The results indicated that, although there was a significant main effect of the type of human–machine interaction on arousal, *F*(2,463) = 3.25, *p* = 0.040, and η_p_^2^ = 0.01, there was no significant interaction between the direction and type of human–machine interaction on arousal, *F*(2,463) = 0.11, *p* = 0.899, and η_p_^2^ = 0.00. This implies that the additional variable of emotion can be entirely ruled out as having an influence on the results.

#### 3.4.2. Experiment 2: Emotion

**(1) Three Dimensions of Emotion (Pleasure, Arousal, and Dominance): 2 × 2 Two-Way ANOVA**

To investigate whether emotional state influenced the experimental results, a 2 × 2 two-way ANOVA was conducted on the three dimensions of emotion (pleasure, arousal, and dominance). The results showed that the main effect of the human–human interaction direction on pleasure was significant, *F*(1,309) = 9.14, *p* = 0.003, and η_p_^2^ = 0.03, and the interaction effect between the human–human interaction direction and the type of human–human interaction on pleasure was also significant, *F*(1,309) = 6.79, *p* = 0.010, and η_p_^2^ = 0.02. To eliminate the confounding effects of emotion, pleasure was included as a covariate in the main effects analysis of the RPP task. It was found that this did not affect the existing significance, indicating that the additional variable of emotion can be completely excluded.

## 4. Discussion

### 4.1. Discussion of Experiment 1

#### 4.1.1. Manipulation Check Results Confirm Effective Manipulation of Interaction Degree

**(1) Regarding RMSE (Position Error)**

The results revealed significant main effects of both the interaction direction and interaction type, as well as a significant interaction effect, indicating that the manipulation of different interaction levels was successful. Across all interaction types, the mirror imitation group showed a significantly lower position error than the mirror avoidance group, consistent with the design goal that imitation should produce greater movement similarity. Regarding interaction type, the Joint Improvisation group showed a significantly higher position error than both the Machine Follows Human and Human Follows Machine groups, while the Machine Follows Human group also showed a higher error than the Human Follows Machine group. This pattern is consistent with the expectation that human players are generally more flexible and adaptive, resulting in lower discrepancies when humans followed the virtual player (Llobera et al., 2022; Froese et al., 2024). Notably, although the Joint Improvisation and Machine Follows Human groups used the same program, the Joint Improvisation group produced a higher position error. This likely reflects the bidirectional demands of joint interaction (Schiavio et al., 2024; Green et al., 2024), in which both agents actively imitate or avoid each other, increasing hesitation and coordination delays, and thereby producing larger positional discrepancies.

**(2) Regarding CV (Phase Stability)**

The results showed no significant main effects of interaction direction or interaction type nor a significant interaction effect. This finding is consistent with the experimental design. Although differences were observed in the position error (RMSE), phase stability remained relatively constant across conditions. A possible explanation is that participants were able to adapt their behavior to maintain a stable level of phase synchronization under different interaction modes (Feniger-Schaal et al., 2021; Werner, 2024). This suggests that participants in all groups were actively engaged with the virtual player, likely supported by prior practice and clear experimental instructions.

#### 4.1.2. Synergistic Effects of Embodied and Enactive Metaphors: Interactive Effects of Human–Machine Interaction Direction and Type on Creativity Dimensions

**(1) Interaction Direction and Embodied Metaphor Mechanisms**

The absence of a significant main effect of interaction direction suggests that predictions derived from embodied metaphor theory, cognitive load theory, and dual-process theory were not fully supported in the present context (Lakoff & Johnson, 1980; Plass et al., 2010; Kahneman, 2011). One possible explanation is that the interaction direction alone may not be a sufficiently strong or independent driver of cognitive processing during creative tasks (Drury & Tudor, 2024; Green et al., 2024). In dynamic human–machine contexts, the effects of embodied metaphors are likely contingent on broader interaction structures, such as role organization and coordination patterns rather than isolated movement patterns. From the perspective of embodied metaphor theory, mirror mimicry may evoke congruent metaphors such as harmony and coordination, which are associated with reduced cognitive load and smoother processing. By contrast, mirror avoidance may induce more incongruent metaphors that disrupt established cognitive patterns and encourage exploratory processing. However, because participants continuously adapted to the virtual partner, these effects may have been attenuated, thereby reducing the clear main effects of interaction direction. Similarly, although dual-process theory suggests that congruent conditions promote analytical processing whereas incongruent conditions facilitate intuitive processing, both processes may have operated simultaneously in socially embedded tasks due to ongoing interpersonal adjustment.

**(2) Interaction Effects and the Unique Role of Joint Interaction Type**

Analysis of the interaction between direction and type revealed significant effects across multiple creativity dimensions. For fluency, joint interaction under both mirror mimicry and avoidance showed high engagement. Sustained mimicry between humans and machines may create a cooperative state that enhances fluency by reducing the cognitive load (Plass et al., 2010; Kolfschoten & Brazier, 2013; Pelkey, 2023). For flexibility, when the machine mimicked the participant, it functioned as a supportive partner, strengthening collaboration and promoting flexibility (Fischer & Amabile, 2009; Yuan et al., 2018). For persistence, under mirror mimicry, persistence was lower in the machine-following-human condition than in the human-following-machine condition, while the latter exceeded the joint interaction group. Lower self-efficacy in the human-following-machine condition may have narrowed the category focus and increased persistence. Under mirror avoidance, persistence was higher in the machine-following-human condition, possibly due to the stronger perceived social presence and responsiveness of the machine (Feniger-Schaal et al., 2021; Li & Zhai, 2022). For novelty, under mimicry, novelty was highest in the machine-following-human condition, whereas under avoidance, the joint interaction group showed the highest novelty. This pattern suggests that incongruent embodied metaphors combined with active engagement may promote novelty.

Overall, the findings highlight the synergistic effects of embodied and enactive metaphors across creativity dimensions and the unique role of joint interaction in human–machine collaboration. Embodied metaphors grounded in bodily movement may enhance simpler dimensions such as fluency and persistence through a consistency effect. However, they appear less effective in explaining flexibility and novelty. Enactive metaphors, based on dynamic interaction and real-time sense-making, help address this limitation (Varela et al., 1991; Thompson & Stapleton, 2009; Fuchs & De Jaegher, 2009; Froese et al., 2024). The incongruence effect in mirror avoidance may disrupt existing cognitive frameworks and expand the exploratory space, thereby enhancing flexibility and novelty. These findings support a more dynamic and social account of creativity within enactive cognition frameworks (Gallagher, 2017; Anderson, 2018; Green et al., 2024).

### 4.2. Discussion of Experiment 2

#### Embodied and Enactive Metaphors Synergistically Enhance Group Creativity: Multidimensional Effects of Interaction Direction and Type on Group Creativity

**(1) Non-Significant Main Effects: The General Influence of Interaction Modes on Group Creativity in Adolescents**

The absence of significant main effects for the interaction direction and interaction type reveals an intriguing phenomenon: for adolescents, both mimicry and avoidance in interaction direction, as well as leader–follower and Joint Improvisation in interaction type, may effectively support group creativity. This finding is broadly consistent with the developmental characteristics of adolescence, a period marked by rapid growth in social skills, cooperation, and peer coordination. Diverse interaction modes may therefore function as catalysts for collaborative creativity. Previous research has similarly shown that team interaction plays an important role in fostering group creativity among adolescents (Paulus, 2000; van der Zanden et al., 2020; Zhao et al., 2022; Green et al., 2024).

From the perspective of embodied metaphor theory, mirror mimicry may evoke congruent metaphors such as harmony and coordination, whereas mirror avoidance may create more incongruent experiences involving conflict and tension. In adolescent groups, however, both forms of bodily interaction may stimulate idea generation in different ways, thereby reducing the likelihood of simple main effects. Likewise, from the perspective of enactive cognition, interaction type may regulate autonomy and control distribution. Joint Improvisation allows for greater spontaneity and real-time co-adaptation, whereas leader–follower structures provide clearer organization and direction. For adolescent participants, both structures may offer functional advantages depending on task demands, which may further explain the absence of general main effects.

**(2) Significant Interaction Effects: The Advantages of the Leader–Follower Model**

The results reveal a clear synergistic advantage of the mirror interaction direction and leader–follower model across multiple dimensions of the RPP task.

For fluency, under mirror avoidance, the leader–follower dyad outperformed Joint Improvisation, likely because cognitive conflict stimulated active exploration while clear role distribution facilitated efficient task decomposition. For flexibility, under mirror mimicry, the leader–follower dyad showed a higher performance, suggesting that consistency effects enhanced collaboration and multi-perspective integration under guided coordination. For persistence, under mirror avoidance, the leader–follower dyad again showed superior performance. The incongruence effect may have promoted continuous strategy adjustment, while role differentiation helped allocate cognitive resources under higher task demands. For novelty, under mirror mimicry, the leader–follower dyad outperformed Joint Improvisation, indicating that coordinated consistency may support novel idea generation within a structured framework. For idea integration, under mirror avoidance, leader–follower pairs showed higher integration, suggesting that cognitive conflict encouraged deeper engagement with partners’ ideas.

Overall, the leader–follower model appears to enhance group creativity through clear role distribution and interdependent coordination. This interpretation is consistent with developmental research indicating adolescents’ sensitivity to structured roles and authority relationships (Qu et al., 2017; Zhao et al., 2022). Compared with Joint Improvisation, the leader–follower model may reduce cognitive load and support more efficient collaboration through organized coordination and dynamic sense-making. These findings further illustrate the synergy between embodied and enactive metaphors in group creativity and provide practical implications for optimizing collaborative processes in educational and organizational settings.

### 4.3. General Discussion

Traditionally, creativity has been commonly defined from the perspective of divergent thinking as the ability to generate novel and appropriate ideas (Amabile, 1983), typically assessed through fluency, flexibility, and novelty (Runco & Jaeger, 2012). To explain complex processes such as creativity, embodied cognition introduced the embodied metaphor theory, which proposes that abstract concepts can be activated through bodily experience (Gibbs et al., 2004; Barrett & Stout, 2024). However, despite its success in explaining lower-order cognition (e.g., perception and action), embodied cognition still faces challenges in accounting for higher-order processes such as creativity (Borghi et al., 2017; Gallagher, 2023).

#### 4.3.1. Challenges in Embodied Creativity

Traditional studies on embodied creativity often adopt a “physical task–static embodied metaphor–creativity task” design. Participants first perform a specific bodily task, followed by an assessment of creative performance. The dominant explanation is based on embodied metaphor theory, which proposes that bodily actions activate corresponding metaphors and thereby facilitate higher-order cognition (Frith & Miller, 2024). For example, Wilson and Gibbs (2007) found that body rotation, associated with “changing perspective,” enhanced flexibility in a semantic association task. Slepian and Ambady (2012) showed that arm extension, linked to “thinking expansively,” improved divergent thinking. Leung et al. (2012) further reported that free walking (“thinking outside the box”), arm switching (“thinking from a different angle”), and coordinated arm movements (“integrated thinking”) all enhanced fluency and flexibility. Similarly, Oppezzo and Schwartz (2014) found that treadmill walking improved performance on the Alternative Uses Task, while Richard et al. (2023) further highlighted the role of bodily movement and embodied action in creative processes. Collectively, these findings suggest that bodily actions can promote creative thinking through embodied metaphors, highlighting the close relationship between bodily experiences and higher-order cognition (Malinin, 2025).

#### 4.3.2. Extending Previous Mirror Game Research

Previous studies have established the mirror game paradigm as a valuable framework for examining bodily coordination and interpersonal interaction (Noy et al., 2011). By contrasting leader–follower and Joint Improvisation conditions, the paradigm captures different coordination structures, ranging from role-based tracking to spontaneous co-regulation. Interaction quality has commonly been assessed through indices of synchrony, temporal coupling, and movement complexity. Research has further shown that movement synchrony is closely related to improvisational creativity, with synchrony often playing a more important role than movement complexity in the development of creative abilities (Gueugnon et al., 2016; Keisari et al., 2022). Subsequent work also expanded the mirror game into human–machine contexts. Computational adaptations enabled coordinated interaction between virtual players and human participants, demonstrating that social motor coordination can be modeled algorithmically and used to generate adaptive, human-like movement patterns (Zhai et al., 2014, 2018; Llobera et al., 2022). These developments suggested that the mirror game could serve not only as a tool for studying human interaction but also as a platform for investigating creativity in technologically mediated settings.

The present findings extend this literature in two important ways. First, Experiment 1 adapted the mirror game to a human–machine context and demonstrated that interaction direction and interaction type jointly influenced multiple dimensions of individual creativity. This suggests that even avatar-based coordination can shape creative cognition, particularly when the interaction structure and movement direction are considered simultaneously. Second, previous studies extended the mirror game to human–human contexts and showed that bodily interaction patterns can reveal meaningful social and relational tendencies, including attachment orientation and nonverbal interaction quality (Feniger-Schaal & Lotan, 2017; Feniger-Schaal et al., 2018, 2021). Building on this line of work, Experiment 2 showed that similar interaction manipulations also influenced group creativity. Significant interaction effects indicate that natural interpersonal coordination may play a stronger role in flexibility and novelty, consistent with prior work emphasizing the importance of embodied social interaction.

Together, these results broaden previous mirror game research from coordination, attachment, and synchrony processes to multidimensional creativity, and support the paradigm as a useful tool for investigating how bodily interaction shapes both individual and group creative performances.

#### 4.3.3. The Dual Mechanisms of Embodied and Enactive Metaphors—Interpreting Creativity from Human–Machine Interaction to Human–Human Interaction

**(1) Theoretical Advances in Embodied Creativity: Dual Mechanisms of Embodied and Enactive Metaphors**

From the perspective of the embodied metaphor theory, interaction direction may shape metaphor generation during creative tasks. Mirror mimicry may evoke synchronization-related metaphors (e.g., harmony and coordination), whereas avoidance may induce more incongruent metaphors that disrupt established cognitive patterns. These contrasting bodily experiences may partly explain why different interaction directions were associated with distinct creativity outcomes across the two experiments.

With the development of embodied cognition, some scholars have argued that the embodied metaphor theory is less radical than initially assumed and remains compatible with traditional representational computational models (Shapiro, 2019; Drury & Tudor, 2024). Although it emphasizes the role of the body in cognition, this does not contradict computational frameworks, aligning with Gallagher’s (2015) concept of “minimal embodiment” (Gallagher, 2023).

To address these limitations, embodied cognition has evolved into the “4E Cognition” framework—embodied, extended, embedded, and enactive cognition (Newen et al., 2018; Su & Ye, 2023). Among these, enactive cognition highlights dynamic interactions between the body, environment, and agents as the basis of sense-making (Malinin, 2019; Werner, 2024). Integrating the metaphor theory with enactive cognition suggests that creativity emerges not from isolated mental processes but from continuous interactions among actions and the environment (Green et al., 2024).

Experiment 1 demonstrates the key role of embodied metaphors in structured human–machine interaction, particularly in enhancing fluency and persistence. Under mimicry conditions, the “consistency effect” reduced cognitive load and stabilized attention, thereby facilitating a lower-order creative performance. However, the limited adaptability of human–machine interaction constrained enactive processes, making it difficult to account for higher-order dimensions such as flexibility and novelty.

In contrast, Experiment 2 highlights the central role of enactive metaphors in human–human interaction, especially in improving flexibility and novelty. The “incongruence effect” under avoidance conditions disrupted existing cognitive frameworks and promoted real-time meaning construction. Under mimicry conditions, the leader–follower structure provided coordinated yet differentiated roles, supporting the generation of novel ideas. These findings indicate that enactive metaphors, through dynamic interaction and adaptive coordination, are crucial for higher-order creativity in complex collaborative contexts.

**(2) From “Metaphor” to “Sense-making”: The Extension of Metaphor Theory Through Enactive Cognition**

Meanwhile, the interaction type may regulate autonomy and control through enactive mechanisms. High-autonomy interactions such as Joint Improvisation may promote real-time adaptation and creative responses, whereas low-autonomy structures such as leader–follower conditions may enhance coordination but constrain expression. This pattern is broadly consistent with the present findings, particularly the stronger flexibility and novelty effects observed under more interactive conditions.

To compare human–machine and human–human interactions, this study sought to maintain consistency in design and procedures across Experiments 1 and 2. However, fundamental differences in interaction naturalness, response speed, and task types (AUT vs. RPP) prevented full standardization. Therefore, the two experiments should be interpreted complementarily rather than directly compared. This distinction highlights “sense-making” as a core concept of enactive cognition: human–machine interaction relies more on the “consistency effect” of embodied metaphors, whereas human–human interaction fosters higher creativity through dynamic sense-making. This provides guidance for improving comparability in future research.

Enactive cognition conceptualizes sense-making as arising from dynamic coupling between the body and environment. According to the “participatory sense-making” framework (De Jaegher & Di Paolo, 2007; Werner, 2024), social interaction involves coordinated action and mutual regulation, enabling the emergence of shared meaning beyond individual capacity. In human–machine interaction, limited machine autonomy constrains participation in this process, leading creativity to manifest primarily in lower-order dimensions (e.g., fluency and persistence). In contrast, human–human interaction supports the real-time co-construction of goals and cognitive frameworks, yielding advantages in higher-order dimensions such as flexibility and novelty.

From this perspective, embodied metaphors and sense-making are deeply interconnected. Jensen and Cuffari (2014), from a radical enactivist and distributed cognition perspective, argue that metaphors are multi-agent, multi-level, and temporally distributed processes grounded in embodied interaction (Drury & Tudor, 2024). Their case analyses demonstrate how metaphoricity emerges through coordinated bodily, verbal, and contextual dynamics, emphasizing its role in joint meaning construction. Similarly, Anderson (2018) shows that embodied metaphors, combined with affective engagement, facilitate deep learning and creative thinking by enabling learners to construct and internalize meaning through active participation (Pelkey, 2023; Schiavio et al., 2024). Together, these perspectives reconceptualize metaphors not as static representations but as dynamic processes of social sense-making, reinforcing their central role in creativity.

#### 4.3.4. Contributions to the Literature

This study contributes to the rapidly evolving literature on embodied cognition and creativity by situating its findings within recent developments that emphasize interaction, social embeddedness, and 4E cognition frameworks. First, it advances theory by proposing a dual-mechanism framework integrating embodied and enactive metaphors, addressing a key limitation in recent research. While contemporary studies increasingly conceptualize creativity as an embodied, situated, and interaction-driven process (e.g., Gallagher, 2023; Frith & Miller, 2024; Malinin, 2025), they typically examine either embodied processes or dynamic interaction in relative isolation. By showing that embodied and enactive metaphors may operate through complementary pathways—supporting lower-order (fluency, persistence) and higher-order (flexibility, novelty) creativity, respectively—this study offers a more fine-grained, process-oriented account within the 4E cognition paradigm. Second, the study contributes methodologically and empirically by introducing a dynamic mirror game paradigm that captures real-time interpersonal coupling. In response to recent calls to move beyond decontextualized laboratory tasks toward socially situated, interaction-based, and multimodal approaches to creativity and learning (e.g., Walkington et al., 2024; Whitehead et al., 2024; Carbajal & Baranauskas, 2025), the present design systematically manipulates the interaction direction and type, enabling the observation of how creativity emerges through coordinated bodily interaction. It further distinguishes between human–machine and human–human interaction mechanisms, showing that the former primarily elicits embodied metaphor effects, whereas the latter more strongly supports enactive sense-making processes. This distinction also speaks to emerging discussions of human–AI collaboration and hybrid intelligence (e.g., Cui & Yasseri, 2024; Noller, 2025), particularly by suggesting that artificial systems still have a limited capacity for genuinely participatory sense-making. Finally, the study contributes to broader creativity research by reconceptualizing creativity as a relational and interaction-driven process rather than merely an individual cognitive capacity. This perspective aligns with recent embodied and socially situated accounts of creativity (e.g., Frith & Miller, 2024; Malinin, 2025), while extending them through direct experimental evidence from embodied interaction contexts and showing how creativity can emerge from dynamic coordination, conflict, and coupling between agents.

#### 4.3.5. Limitations

Although embodied cognition emphasizes the role of the body in cognition, its dominant approach of transforming external stimuli into embodied forms still largely operates within a symbolic representational framework (Shapiro, 2019; Drury & Tudor, 2024). For example, the “source domain → target domain” mapping in embodied metaphor theory remains a form of symbolic translation, which may limit its capacity to explain higher-order cognition such as creativity. Moreover, although the static embodied metaphor paradigm has received empirical support, it also presents several limitations relevant to the present study. First, its effects are often context-dependent and may be difficult to reproduce, as metaphor interpretations can vary across cultural and experimental settings. Second, its underlying mechanisms often lack precise operational definitions and may rely on experiential assumptions. Finally, this paradigm is largely constrained by a linear stimulus–response model, which may overlook the role of dynamic interaction in higher-order cognition and the complexity of real-time metaphor generation.

In Experiment 1, the virtual player was implemented using the Human Dynamic Clamp (HDC) framework to enable real-time bidirectional human–machine interaction. However, the present study did not incorporate the framework proposed by Li and Zhai (2022), which still has certain limitations in simulating or guiding intentional coupling processes and individual movement characteristics. Future research could further integrate related models to enhance the flexibility and precision of human–machine interaction. In addition, this study modeled two-dimensional interactive motion using Lissajous figures, generating periodic trajectories through orthogonal harmonic motion. Nevertheless, this approach remains limited in terms of dynamic complexity, as trajectory variations were primarily restricted to adjustments in frequency and amplitude. Future studies could extend this modeling approach to three-dimensional or higher-complexity interaction paradigms to improve ecological validity.

In Experiment 2, there is currently a lack of well-established methods for quantifying human–human interaction, which may constrain the fine-grained analysis of interaction processes. Future research could incorporate computer vision techniques and automated analysis methods to achieve more objective measurements of bodily interaction. For example, Feniger-Schaal et al. (2021) assessed movement synchrony in mirror game interactions using motion energy analysis and time-series algorithms, providing a valuable methodological reference for interaction research.

In addition, multiple analyses of variance (ANOVAs) and post hoc comparisons were conducted in this study without applying formal corrections for multiple comparisons (e.g., Bonferroni or FDR correction), which may increase the risk of a Type I error. Therefore, results with small effect sizes or marginal significance should be interpreted with caution.

#### 4.3.6. Future Directions: Could Predictive Processing Explain How Bodily Interaction Enhances Creativity?

While enactive cognition offers innovative perspectives on higher-order cognitive processes such as creativity, its explanation of the specific mechanisms linking bodily interaction to these processes—particularly the roles of metaphor and sense-making—remains ambiguous. This suggests a need for further development of the enactive cognition theory, potentially through integration with predictive processing models (Clark, 2016). Predictive processing models align closely with enactive cognition in their emphasis on cognition as the product of dynamic interactions between individuals and their environment rather than passive information processing. Predictive processing focuses on minimizing prediction errors through feedback loops between top-down predictions and bottom-up sensory input, optimizing cognitive processes. This framework could provide enactive cognition with formalized and mathematical support. By combining prediction error minimization with dynamic sense-making in enactive metaphors, future research could better explain how bodily interaction enhances creativity by optimizing feedback loops or reducing cognitive load. Specifically, future research may proceed in two directions. **(1) Exploring the evolutionary–predictive algorithm** by building on Dietrich and Haider (2015) to examine how variation–selection coupling contributes to creative thinking. **(2) Integrating active inference frameworks** by applying Lehmann et al. (2022)’s model to investigate how predictive adjustment and real-time feedback enhance team creativity.

## 5. Conclusions

This study examined how bodily interaction direction and interaction type influence creativity across human–machine and human–human mirror game paradigms. Overall, the findings indicate that creativity is shaped less by either factor alone than by their combined effects. In Experiment 1, significant interaction effects between the human–machine interaction direction and type were found for fluency, flexibility, persistence, and novelty in the Alternative Uses Task. These results suggest that different forms of avatar interaction jointly influence individual creativity. In Experiment 2, no significant main effects of the interaction direction or interaction type were found for group creativity dimensions in the person-to-person setting. However, their interaction significantly influenced group creativity performance in the RPP task, although no significant effect was observed for idea integration. Taken together, the results provide partial support for H1 and H2, namely, that bodily interaction direction and bodily interaction type may influence multiple dimensions of creativity. However, the findings provide stronger and more consistent support for H3, indicating that bodily interaction direction and type primarily shape creativity through their interaction rather than through independent main effects. These findings highlight the importance of dynamic bodily coordination in creativity and support an integrated embodied–enactive account of creative cognition.

## Figures and Tables

**Table 1:** Descriptive statistics for interaction degree: RMSE and CV.

Interaction Level	Human–Machine Interaction Direction	Human–Machine Interaction Type	*M*	*SD*
RMSE (Position Error)	Mirror Mimicry	Machine Follows Human (LF)	0.40	0.29
		Human Follows Machine (LF)	0.10	0.04
		Joint Improvisation (JI)	0.54	0.16
	Mirror Avoidance	Machine Follows Human (LF)	0.68	0.24
		Human Follows Machine (LF)	0.62	0.22
		Joint Improvisation (JI)	0.80	0.16
CV (Phase Stability)	Mirror Mimicry	Machine Follows Human (LF)	0.01	0.01
		Human Follows Machine (LF)	0.01	0.01
		Joint Improvisation (JI)	0.01	0.01
	Mirror Avoidance	Machine Follows Human (LF)	0.02	0.05
		Human Follows Machine (LF)	0.01	0.01
		Joint Improvisation (JI)	0.01	0.01

**Table 2:** Inter-rater reliability results of two independent raters for the AUT.

Scoring Dimension	AUT	Cronbach’s Alpha
Flexibility	Chopsticks	0.804
	Key	0.821
Persistence	Chopsticks	0.890
	Key	0.840
Novelty	Chopsticks	0.710
	Key	0.683

**Table 3:** Inter-rater reliability results of two independent raters for the RPP task.

Rating Dimension	RPP Task	Cronbach’s Alpha
Dyadic Flexibility	Desk-mate Disturbance	0.958
Dyadic Persistence	Desk-mate Disturbance	0.946
Dyadic Novelty	Desk-mate Disturbance	0.899
Dyadic Integration of Ideas	Desk-mate Disturbance	0.890

**Table 4:** Descriptive statistics for performance on the AUT chopstick task.

Human–Machine Interaction Direction	Human–Machine Interaction Type	Chopstick Fluency		Chopstick Flexibility		Chopstick Persistence		Chopstick Novelty	
		*M*	*SD*	*M*	*SD*	*M*	*SD*	*M*	*SD*
Mirror Imitation	Machine Follows Human (LF)	15.48	8.39	5.22	1.50	2.86	1.27	4.17	1.40
	Human Follows Machine (LF)	15.30	6.82	4.81	1.21	3.26	1.65	3.60	1.40
	Joint Improvisation (JI)	12.19	5.86	3.85	1.11	3.10	1.13	3.76	1.32
Mirror Avoidance	Machine Follows Human (LF)	16.81	7.65	4.55	1.43	3.76	1.58	4.03	1.43
	Human Follows Machine (LF)	14.26	8.05	4.57	1.42	3.04	1.75	3.74	1.54
	Joint Improvisation (JI)	17.20	7.87	4.21	1.46	4.25	2.12	4.33	1.49

**Table 5:** Descriptive statistics for performance on the AUT key task.

Human–Machine Interaction Direction	Human–Machine Interaction Type	Key Fluency		Key Flexibility		Key Persistence		Key Novelty	
		*M*	*SD*	*M*	*SD*	*M*	*SD*	*M*	*SD*
Mirror Imitation	Machine Follows Human (LF)	14.02	19.36	3.73	1.65	3.54	3.34	3.33	1.42
	Human Follows Machine (LF)	13.13	6.64	3.37	0.95	3.98	2.14	2.86	1.06
	Joint Improvisation (JI)	11.01	5.79	3.15	1.12	3.50	1.44	3.35	1.24
Mirror Avoidance	Machine Follows Human (LF)	14.81	7.58	3.36	1.19	4.90	3.99	3.10	1.31
	Human Follows Machine (LF)	9.80	6.05	3.11	1.28	3.21	2.56	2.73	1.26
	Joint Improvisation (JI)	14.76	7.66	3.60	1.36	4.26	2.33	3.58	1.21

**Table 6:** Descriptive statistics for the RPP task (dyadic fluency, flexibility, novelty, persistence, and viewpoint integration).

Human–Human Interaction Direction	Human–Human Interaction Type	RPP Fluency		RPP Flexibility		RPP Persistence		RPP Novelty		Integration of Ideas	
		*M*	*SD*	*M*	*SD*	*M*	*SD*	*M*	*SD*	*M*	*SD*
Mirror Imitation	Leader–Follower (LF)	16.31	7.77	8.67	3.76	4.77	4.36	10.25	3.92	1.71	1.54
	Joint Improvisation (JI)	16.65	6.76	7.02	3.00	6.07	5.34	8.35	3.45	2.27	1.50
Mirror Avoidance	Leader–Follower (LF)	17.96	6.21	6.67	2.72	6.86	4.18	8.13	3.48	2.46	1.47
	Joint Improvisation (JI)	14.15	5.80	7.56	3.47	4.24	1.78	9.65	3.55	2.28	1.66

## Data Availability

None of the experiments were preregistered. The data supporting the findings of this study are hosted on the Open Science Framework (OSF) platform. However, access to the data is restricted to safeguard participant privacy. Researchers interested in accessing the dataset may contact the first author (Jiajia Su) to request permission. Data will be shared upon reasonable request and approval.
